# A semidominant point mutation of Mediator tail subunit MED5b in Arabidopsis leads to altered enrichment of H3K27me3 and reduced expression of targets of MYC2

**DOI:** 10.1093/g3journal/jkae301

**Published:** 2025-02-14

**Authors:** Jiaxin Long, Shelby Sliger, Zhi-Wei Luo, Pete E Pascuzzi, Clint Chapple, Joe Ogas

**Affiliations:** Department of Biochemistry, Purdue University, West Lafayette, IN 47906, USA; Department of Biochemistry, Purdue University, West Lafayette, IN 47906, USA; Department of Biochemistry, Purdue University, West Lafayette, IN 47906, USA; Department of Biochemistry, Purdue University, West Lafayette, IN 47906, USA; Purdue University Libraries and School of Information Studies, Purdue University, West Lafayette, IN 47907, USA; Department of Biochemistry, Purdue University, West Lafayette, IN 47906, USA; Purdue University Center for Plant Biology, Purdue University, West Lafayette, IN 47907, USA; Department of Biochemistry, Purdue University, West Lafayette, IN 47906, USA; Purdue University Center for Plant Biology, Purdue University, West Lafayette, IN 47907, USA

**Keywords:** Mediator complex, *ref4-3*, MYC2, H3K27me3 homeostasis, environmental response, *Arabidopsis thaliana*, Plant Genetics and Genomics

## Abstract

The Mediator complex coordinates regulatory input for transcription driven by RNA polymerase II in eukaryotes. *reduced epidermal fluorescence4-3* (*ref4-3*) is a semidominant mutation that results in a single amino acid substitution in the Mediator tail subunit Med5b. Previous characterization of *ref4-3* revealed altered expression of a variety of loci in Arabidopsis, including those contributing to phenylpropanoid biosynthesis. Examination of existing RNA-seq data indicated that loci enriched for the transcriptionally repressive chromatin modification H3K27me3 are overrepresented among genes that are misregulated in *ref4-3*. We used ChIP-seq and RNA-seq to examine the possibility that perturbation of H3K27me3 homeostasis in *ref4-3* plants contributed to altered transcript levels. We observed that *ref4-3* results in a modest global reduction of H3K27me3 at enriched loci and that this reduction is not dependent on gene expression; however, altered H3K27me3 was not strongly predictive of altered expression in *ref4-3* plants. Instead, our analyses revealed a substantial enrichment of targets of the MYC2 transcriptional regulator among genes that exhibit decreased expression in *ref4-3*. Consistent with previous characterization of *ref4-3*, we observed that *ref4-3*-dependent decreased expression of MYC2 targets can be suppressed by loss of another Mediator tail subunit, MED25. This observation is consistent with previous biochemical characterization of MYC2. Our data highlight the diverse and distinct impacts that a single amino acid change in the tail subunit of Mediator can have on transcriptional circuits and raise the prospect that Mediator directly contributes to H3K27me3 homeostasis in plants.

## Introduction

Mediator is a conserved modular complex that coordinates regulatory information conveyed by transcription factors to RNA polymerase II (RNAPII) and is also a component of the preinitiation complex (PIC) that initiates RNAPII-driven transcription (Dolan and Chapple 2017; Malik *et al*. 2017; Chen *et al*. 2022; Richter *et al*. 2022). As such, it plays a critical role in integrating the diverse regulatory pathways that govern transcription at a multitude of loci in eukaryotes. Phylogenetic and structural analysis of Mediator in yeast and mammals reveals substantial evolution of this conserved complex. There are 21 Mediator subunits in the budding yeast *Saccharomyces cerevisiae* and 26 subunits in humans. Cryo-EM structural analysis in yeast (Robinson *et al*. 2015) and subsequently in humans (Abdella *et al*. 2021) and mice (Zhao *et al*. 2021) reveals 4 comparable modules: a head module, a middle module, a tail module, and a kinase module (Dotson *et al*. 2000; Richter *et al*. 2022). The structure of the yeast and vertebrate Mediator complexes are clearly distinguishable, however, consistent with the emergence of novel regulatory strategies for Mediator in these phyla. Further, although depletion or ablation of subunits in yeast indicates that the individual modules function independently in this organism, extensive interconnections between subunits of Mediator in human and mouse (Richter *et al*. 2022) suggest that the modules function in a more coordinated fashion in mammals.

In both yeast and animals, the 4 subunits of Mediator play distinct roles. The kinase module reversibly associates with the other 3 modules of Mediator to generate 2 distinct Mediator complexes, sometimes referred to as Mediator and CDK-Mediator, that have different regulatory properties (Luyties and Taatjes 2022). Association of the kinase module with Mediator blocks its interaction with RNAPII and thus assembly of PIC. Yet the kinase module also contributes to promoter escape of RNAPII and release of RNAPII from pausing during elongation (Luyties and Taatjes 2022), demonstrating the dynamic nature of Mediator during the various stages of transcription. The head and the middle module of mediator interact with RNAPII and general transcription factors directly, whereas the tail module recognizes gene-specific TFs and integrates signals from TFs for transcriptional regulation (Allen and Taatjes 2015; Yang *et al*. 2016; Chen *et al*. 2022; Richter *et al*. 2022).

Although the structure of a plant Mediator complex has yet to be reported, phylogenetic, genetic, and biochemical analyses to date are largely consistent with previous characterization of Mediator in yeast and animals (Buendia-Monreal and Gillmor 2016; Yang *et al*. 2016; Dolan and Chapple 2017; Malik *et al*. 2017; Agrawal *et al*. 2021; Leydon *et al*. 2021). Functional analysis of an extensive collection of mutations in Mediator subunits in Arabidopsis has linked Mediator to a wide range of transcriptional pathways in plants including those involved in abiotic and biotic stress, hormone signaling, development, and metabolism. Analysis of these mutants has also linked specific modules to transcriptional pathways in plants. Functional analysis of MED12 and MED13 of the kinase module, for example, links the kinase module to repression of seed maturation genes during early seedling growth (Chhun *et al*. 2016) as well as to activation of light-responsive genes and genes lacking chromatin features associated with robust expression (Liu *et al*. 2020).

Genetic characterization of the tail module of Mediator in Arabidopsis suggests a critical role in conferring regulatory information by transcription factors (Buendia-Monreal and Gillmor 2016; Yang *et al*. 2016; Mao *et al*. 2019b; Zhai *et al*. 2020; Chen *et al*. 2022). Mutations in tail subunits impact a wide variety of plant adaptive responses including plant defense, jasmonic acid (JA) signaling, phenylpropanoid biosynthesis, as well as cold and shade responses. Biochemical characterization of MED25 has identified over 20 transcription factors with which it interacts (Yang *et al*. 2016; Kazan 2017; Zhai *et al*. 2020) including MYC2 (Cevik *et al*. 2012; Chen *et al*. 2012; An *et al*. 2017; Zhai *et al*. 2020), strongly suggesting that this regulatory information is conveyed by direct interaction of transcription factors with tail subunits, contributing to either transcriptional activation or repression.

Previous characterization of *MED5a* and *MED5b* of the tail module of Mediator in Arabidopsis links both to homeostasis of transcriptional circuits associated with phenylpropanoid metabolism (Bonawitz *et al*. 2012). *med5a med5b* plants exhibit elevated transcript levels of genes associated with phenylpropanoid biosynthesis whereas a semidominant mutant allele of *MED5b*, *ref4-3*, exhibits constitutive repression of genes associated with phenylpropanoid biosynthesis. Intriguingly, mutation of genes encoding other subunits of the tail module, including *MED2*, *MED16*, and *MED23*, suppresses the transcript and metabolic phenotype of *ref4-3*, indicating that an intact tail module is required for *ref4-3* to impair transcriptional regulation (Dolan *et al*. 2017).

We observed that genes enriched for the repressive epigenetic modification trimethylation of histone H3 lysine 27 (H3K27me3) are overrepresented in genes exhibiting altered expression in *ref4-3* plants. To further examine the relationship between *ref4-3* and H3K27me3, we undertook ChIP-seq and RNA-seq. Our data indicate that *ref4-3* results in a reduction of H3K27me3 at many loci, including those with no detectable expression. This loss of H3K27me3 is likely to contribute to previously characterized *ref4-3* phenotypes. Further, our data reveal a dramatic impact on a subset of transcriptional pathways directly mediated by MYC2. Our combined data raise the possibility that Mediator can contribute to chromatin features in a transcription-independent fashion and highlight the impact of changing a single amino acid of a Mediator tail subunit on a transcriptional circuit.

## Materials and methods

### Plant lines and growth conditions

Plants used in this study are all in the Columbia background. The previously characterized mutants used in this study are *ref4-3*, *myc2* (SALK_017005), *myc2 ref4-3*, *med25* (SALK_129555), and *med25 ref4-3*. The genotype of *ref4-3* was confirmed by PCR followed by restriction digestion as described previously (Stout *et al*. 2008). The list of primers used for PCR verification for each allele is included in Supplementary Table 18. Plants used for RNA-seq, ChIP-seq, metabolite MS analysis, and qPCR were sown directly on soil in pots. Seeds were cold treated in dark at 4°C for 3 days before moving to a Percival AR75 growth chamber. Plants were grown under 16-h light/8-h dark photoperiod cycle at 150 μE light (Phillip F32T8/TL941) with far-red supplements (soft white 25 W incandescent light) at 22°C prior to sample collection. Samples for the following analyses were harvested 6 h after the light was turned on.

### RNA extraction

Total RNA was extracted from 21-day-old shoot tissue of 5 plants that had not bolted and that were randomly selected from 5 individual pots using a RNeasy Plant Mini Kit (Qiagen; catalog no. 74904) following with the DNase I digestion and RNA cleanup included in an RNA Clean & Concentrator kit (Zymo Research; catalog no. 1014). Three biological replicates were collected for each indicated genotype.

### Chromatin immunoprecipitation

Chromatin immunoprecipitation was performed as previously described (Carter *et al*. 2018) with minor modifications. Four grams of 21-day-old whole shoot tissue was collected for each replicate. Nuclear lysis buffer was replaced with 1.7 mL of ChIP Lysis Buffer [50 mM HEPES-NaOH (pH 7.4), 16.7 mM Tris-HCl (pH 8), 1 mM EDTA (pH 8), 150 mM NaCl, 0.1% SDS (w/v), 1% Triton X-100 (v/v), 0.1% sodium deoxycholate, 1 cOmplete mini tablet per 10 mL solution]. The total chromatin samples were sonicated using Bioruptor Pico. Sheared chromatin samples were stored at −80°C overnight prior to the immunoprecipitation. The following antibodies were used in the analysis: Anti-H3 (ab1791, Abcam) (1:200 dilution), Anti-H3K27me3 (07-449, Millipore) (1:100 dilution), and Anti-H2A.Z (1.5 µg for a 500 µL IP sample) (Deal *et al*. 2007). The QIAquick MinElute PCR purification kit was replaced with ChIP DNA Clean & Concentrator purification kit (D5205, Zymo Research) for elution of the ChIP DNA. The eluted ChIP DNA was quantified using a Qubit 2.0 Fluorometer and used immediately in library construction.

### Library preparation and sequencing

cDNA library construction and ChIP DNA library construction were performed in house. One microgram of total RNA of each sample was used for cDNA library construction. cDNA library construction was performed using an NEBNext Ultra II Directional RNA Library Prep Kit (NEB; catalog no. E7760S) combined with an NEBNext Poly(A) mRNA Magnetic Isolation Module (NEB; catalog no. E7490S) according to the manufacturer's manual. 0.5 ng eluted ChIP DNA was used for each library construction. ChIP DNA libraries were constructed using a NEBNext Ultra II DNA Library Prep Kit (NEB; catalog no. E7645L) according to the manufacturer's manual. Illumina Multiplexing adapter was used for indexing. Paired-end 150-bp sequencing was performed using an Illumina NovaSeq S4 lane.

### Differential expression analysis

About 70 million reads per sample were trimmed of adapter and polyG sequences using fastp (v 0.23.2) (Chen *et al*. 2018). The quality of the trimmed fastq files was examined using fastQC (v 0.12.1). The resulting fastq files mapped to the TAIR10 reference genome assembly (Pruitt *et al*. 2012) using hisat2 (v 2.2.1). The mapped SAM files were converted to BAM files using SAMtools (v 1.17) (Li *et al*. 2009). The mapped BAM files were sorted and indexed using SAMtools. The gene feature was counted in DESeq2 and limma packages in Bioconductor. Differentially expressed genes (DEGs) were identified using the Bioconductor edgeR package (v 3.42.4) (Robinson *et al*. 2010) with Fisher's exact test with a Benjamini–Hochberg false discovery rate (FDR) threshold of <0.05 and a fold change threshold > 2 (Robinson *et al*. 2010). Genes were annotated based on Araport 11 annotation (Cheng *et al*. 2017).

### Enrichment and differential enrichment analyses

About 70 million reads per sample were trimmed of adapter and polyG sequences using fastp (v 0.23.2) (Chen *et al*. 2018). The quality of the trimmed fastq files was examined using fastQC (v 0.12.1). The trimmed fastq files mapped to the TAIR10 reference genome assembly (Berardini *et al*. 2015) using Bowtie2 (v 2.4.2) (Langmead and Salzberg 2012). The mapped SAM files were converted to BAM files using SAMtools (v 1.17) (Li *et al*. 2009). The resulting BAM files were filtered such that only proper pairs of the reads were kept for the following analysis. The filtered BAM files then sorted and indexed with SAMtools. For peak-finding, the BAM files were converted to paired-end BED files. Epic2 (v 0.0.52) (Stovner and Saetrom 2019) was used for peak-calling with the effective genome size equals to 0.85 and a bin size and gap size equals to 200 bp. There was high correlation between individual replicates (0.8 or greater for H2AZ and 0.9 or greater for H3K27me3), so the BAM files were merged to identify a final set of islands. The identified islands were converted to BED files for following analysis and visualization in IGV.

Identified epic2 islands of each modification in *ref4-3* and wild-type plants were merged by union to determine the islands of interests. Genes marked by the chromatin-associated features were determined by the exhibiting a minimum 200 bp overlaps with the identified epic2 islands. The islands of interests were intersected with genes with a minimum 200 bp overlaps to decide the regions of interests. In general, genes intersected with a single H2AZ or H3K27me3 island, but 11% of identified genes intersected 2 or more H2AZ islands whereas 4% of identified genes intersected with 2 or more H3K27me3 islands. To determine ChIP-seq counts within these regions, the BAM files for each sample were used with the Bioconductor function summarizeOverlaps with the following arguments: mode = ”Union”, inter.feature = FALSE, ignore.strand = TRUE, singleEnd = FALSE, and fragments = FALSE. The resulting count matrix was used for differentially enrichment analysis. Genes that are differentially enriched for H3K27me3 and/or H2A.Z were identified using the Bioconductor edgeR package (v 3.42.4) with Fisher's exact test with a Benjamini–Hochberg FDR threshold of <0.05 and a fold change threshold > 1.2 (Robinson *et al*. 2010). Genes were annotated based on Araport11 annotation (Cheng *et al*. 2017).

### Generation of metagene plots and heatmaps

The metagene plots and heatmaps were generated with deepTools2.0 (v 3.5.1). Indexed BAM files were converted to bigwig files using bamCompare. Reads per kilobase per million mapped reads (RPKM) was used as the normalization method, and the subtract operation was used to normalize the IP sample to its corresponding input DNA. The resulting bigwig files were converted to metagene plots and heatmaps using computeMatrix and plotProfile. Standard error for each metagene plot is indicated by shading of the trendline and was determined using *–plotType se* in the plotProfile function.

### Jasmonic acid extraction

The extraction of JA was performed as previously described in Pan *et al*. (2010) with modifications. Whole shoot tissues of 3-week-old plants grown in different batches were pooled and immediately frozen in liquid nitrogen after harvesting. Leaf tissues were then ground into powder in liquid nitrogen and weighed. Extraction solvent, acidified isopropanol (isopropanol/H_2_O/concentrated HCl, 2:1:0.002, v/v/v), was added at a concentration of 100 mg fresh mass per milliliter along with 50 ng (±)-JA-d_5_ (Cayman Chemical) as the internal standard. Samples were extracted on a shaker at a speed of 15 r.p.m. for 30 min at 4°C. Then 0.55 mL extract was mixed with 1 mL dichloromethane and shaken for an additional 30 min at 4°C. Mixtures were then centrifuged at 13,000×g for 5 min at 4°C. About 900 μL solvent from the lower of 2 phases was transferred to a new tube and concentrated under nitrogen flow. Dried samples were stored at −20°C until analysis.

### Jasmonic acid quantification by LC-MS/MS

Samples were redissolved in 100 μL methanol, and a 10 μL sample was injected into an Agilent 1290 Infinity II liquid chromatography (LC) system coupled to an Agilent 6470 series QQQ mass spectrometer (MS/MS, Agilent Technologies, Santa Clara, CA, USA) to quantify JA levels. For LC separation, an Agilent Eclipse Plus C18 2.1 mm × 50 mm, 1.8 µm column was used. The buffers were (A) water + 0.1% formic acid (v/v) and (B) acetonitrile + 0.1% formic acid. The linear LC gradient was as follows: time 0 min, 5% B; time 1 min, 5% B; time 10 min, 95% B; time 11 min, 95% B; time 11.1 min, 5% B; time 15 min, 5% B. The flow rate was 0.350 mL/min. Multiple reaction monitoring was used for MS analysis. (For JA, a precursor ion 209.2 to a product ion 59.3 transition was monitored at collision energy of 10 V and a transition from 209.2 to 41.3 was monitored at 40 V. For JA-d_5_, a transition from 214.3 to 62.3 was monitored at 10 V and a transition from 214.3 to 42.3 was monitored at 40 V.) Data were acquired in negative electrospray ionization (ESI) mode. The jet stream ESI interface had a gas temperature of 325°C, gas flow rate of 7 L/min, nebulizer pressure of 45 psi, sheath gas temperature of 250°C, sheath gas flow rate of 7 L/min, capillary voltage of 3,500 V in negative mode, and nozzle voltage of 1,000 V. The ΔEMV voltage was 400 V. Agilent Masshunter Quantitative analysis software was used for data analysis (v 10.1).

### cDNA reverse transcription and quantitative real-time PCR analysis

Total RNA of 3 biological replicates of wild-type, *ref4-3*, *myc2*, *myc2 ref4-3*, *med25*, and *med25 ref4-3* plants were collected for quantitative gene expression analysis. Two micrograms of total RNA was used for cDNA reverse transcription with an M-MLV Reverse Transcriptase kit according to the manufacturer's protocol. qPCR was performed using PowerUp SYBR Green Master Mix. The qPCR thermal cycle was performed according to the manufacture's manual. Oligonucleotide primers used for quantification of gene expression were included in Supplementary Table 18. *C*_T_ values were recorded by Applied Biosystems software. 18S was used as the housekeeping gene for normalization. For each biological replicate, there are 4 Δ*C*_T_ values computed by reciprocally subtracting the *C*_T_ values of 18S from the *C*_T_ values of the gene of interest with their corresponding technical replicates. The ΔΔ*C*_T_ values were calculated by subtracting the average Δ*C*_T_ of WT for the gene of interest from the Δ*C*_T_ value of the experimental condition and gene of interest. Fold changes were calculated using the formula 2^−ΔΔCT^. Standard error of the fold changes for each genotype was calculated.

## Results

### *ref4-3* plants exhibit global reduction of H3K27me3

Previous RNA-seq analysis of *ref4-3* plants revealed large numbers of genes with altered transcript levels (Dolan *et al*. 2017; Mao *et al*. 2019a). Given the extensive developmental phenotypes associated with the presence of *ref4-3* (Stout *et al*. 2008; Wang *et al*. 2020), we examined whether genes enriched for the histone modification H3K27me3, which restricts expression of many genes associated with differentiation and development (Bouyer *et al*. 2011; Mozgova and Hennig 2015; Shen *et al*. 2021), are overrepresented in genes exhibiting altered transcript levels in *ref4-3* plants and found this to be the case (Supplementary Fig. 1). This observation raised the possibility that *ref4-3* perturbs H3K27me3 homeostasis and/or H3K27me3-mediated gene repression.

To characterize the effect of *ref4-3* on H3K27me3-associated processes, we undertook ChIP-seq and RNA-seq analysis of wild-type and *ref4-3* plants. In addition to H3K27me3, we also used ChIP-seq to examine enrichment of H2A.Z, a chromatin mark that is also typically found at H3K27me3-marked genes (Sequeira-Mendes *et al*. 2014; Carter *et al*. 2018; Gomez-Zambrano *et al*. 2019). The resulting ChIP-seq data are consistent with previous analyses. We identified 8,286 genes that are marked by H3K27me3 and 22,249 genes that are marked by H2A.Z in either wild-type or *ref4-3* plants (Supplementary Tables 1–4). Eighty-seven percent of previously identified H3K27me3-enriched genes were also identified by our analysis (Zhou *et al*. 2017). Similarly, 80% of previously identified H2A.Z-marked genes were also observed as H2A.Z-marked genes in our analysis (Wollmann *et al*. 2017).

**Fig. 1.**
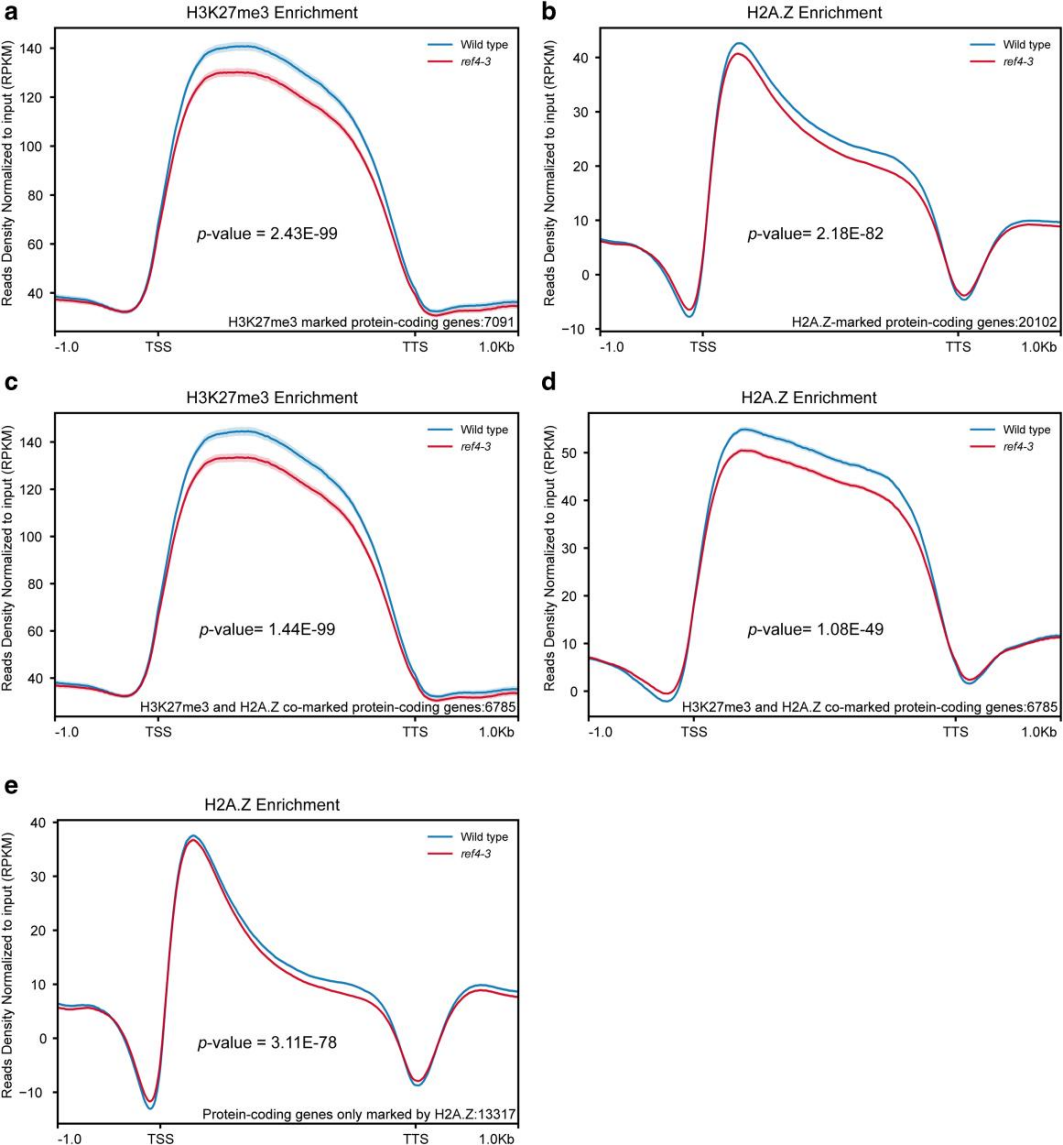
The pattern of H3K27me3 and H2A.Z enrichment is altered in *ref4-3* plants. Metagene profiles derived from current ChIP-seq analysis were used to examine the impact of *ref4-3* on a) average H3K27me3 enrichment level for H3K27me3-marked protein-coding genes; b) average H2A.Z enrichment level for H2A.Z-marked protein-coding genes; c) average H3K27me3 enrichment level from protein-coding genes that are marked by both H3K27me3 and H2A.Z; d) average H2A.Z enrichment level from protein-coding genes that are marked by both H3K27me3 and H2A.Z; and e) average H2A.Z enrichment level from protein-coding genes that are only marked by H2A.Z. In the metagene profiles, RPKM are generated as described in “Materials and methods” and are representative of merged 2 independent biological replicates of each genotype in which the IP sample of the mark of interest is normalized to its corresponding input DNA. RPKM values are plotted with respect to gene regions that are scaled to 2,500 bp on the *x*-axes. The total number of genes that are included in each plot is indicated in the lower right corner. Biological samples were obtained from the shoot tissue of 21-day-old plants grown under a 16-h light/8-h dark photoperiod cycle. The standard error associated with each analyzed genomic region depicted on the trend line is indicated by the shading surrounding each line. In addition, the significance of the difference in the average level of chromatin-associated features in wild-type and *ref4-3* plants was determined by a paired sample *t*-test and is indicated on each plot. TSS, transcription start site; TTS, transcription termination site.

To investigate whether *ref4-3* perturbs the global enrichment of H3K27me3 and/or H2A.Z, we generated metagene plots of H3K27me3 and H2A.Z ChIP-seq signals. The metagene plots of H3K27me3 (Fig. 1a) reveal a modest reduction of H3K27me3 levels for H3K27me3-enriched genes in *ref4-3* plants. We also observed a similarly modest reduction of H2A.Z enrichment at H2A.Z-marked genes in *ref4-3* plants (Fig. 1b). Use of ranked heatmaps to depict these analyses indicates that these trends represent similar effects on most genes rather than larger effects on relatively few genes (Supplementary Fig. 2a and b).

The observation that H3K27me3 is often colocalized with H2A.Z in plants (Sequeira-Mendes *et al*. 2014) in conjunction with other data (Carter *et al*. 2018) raises the prospect that H2A.Z contributes to H3K27me3 homeostasis in some fashion. Consistent with previous analyses, we observed that over 87% of H3K27me3-marked genes are also marked by H2A.Z in wild-type plants (Sequeira-Mendes *et al*. 2014; Carter *et al*. 2018). To characterize how *ref4-3* contributes to enrichment of H3K27me3 and H2A.Z in a similar chromatin context, we examined enrichment of these marks specifically in genes in which both marks are present (Supplementary Table 5). Metagene profiles of the 6,785 protein-coding genes that are enriched for both H3K27me3 and H2A.Z reveal a comparable reduction for both of these features (Fig. 1c and d). In contrast, similar analysis of H2A.Z abundance at genes that are only marked by H2A.Z reveals a much more modest reduction of H2A.Z (but still significant based on standard error and statistical analysis) (Fig. 1e). Ranked heatmaps again indicate that these trends represent similar effects on most genes (Supplementary Fig. 2c–e). These data indicate that *ref4-3* preferentially impacts H2A.Z enrichment in the context of H3K27me3, consistent with the hypothesis that the presence of *ref4-3* perturbs H3K27me3 homeostasis and with the hypothesis that H2A.Z and H3K27me3 are functionally linked in some fashion at H3K27me3-enriched loci.

### Reduction of H3K27me3 in *ref4-3* plants is associated with altered transcript levels and is observed at loci that are not expressed

Our RNA-seq data enabled us to examine the possible contribution of perturbed H3K27me3 enrichment to altered transcript levels in *ref4-3* plants and to address the possibility that *ref4-3* affects H3K27me3 enrichment at some loci in a transcription-independent fashion. Given the association of Mediator with Pol II-transcribed genes, we used a gene-centric approach by applying the RNA-seq-based method edgeR (Robinson *et al*. 2010; Wu *et al*. 2015; Eder and Grebien 2022) to our ChIP-seq analysis. Our intent in using this approach was to examine the relationship between altered levels of the chromatin marks H3K27me3 and H2A.Z and altered transcript levels specifically at transcribed loci of interest.

**Fig. 2.**
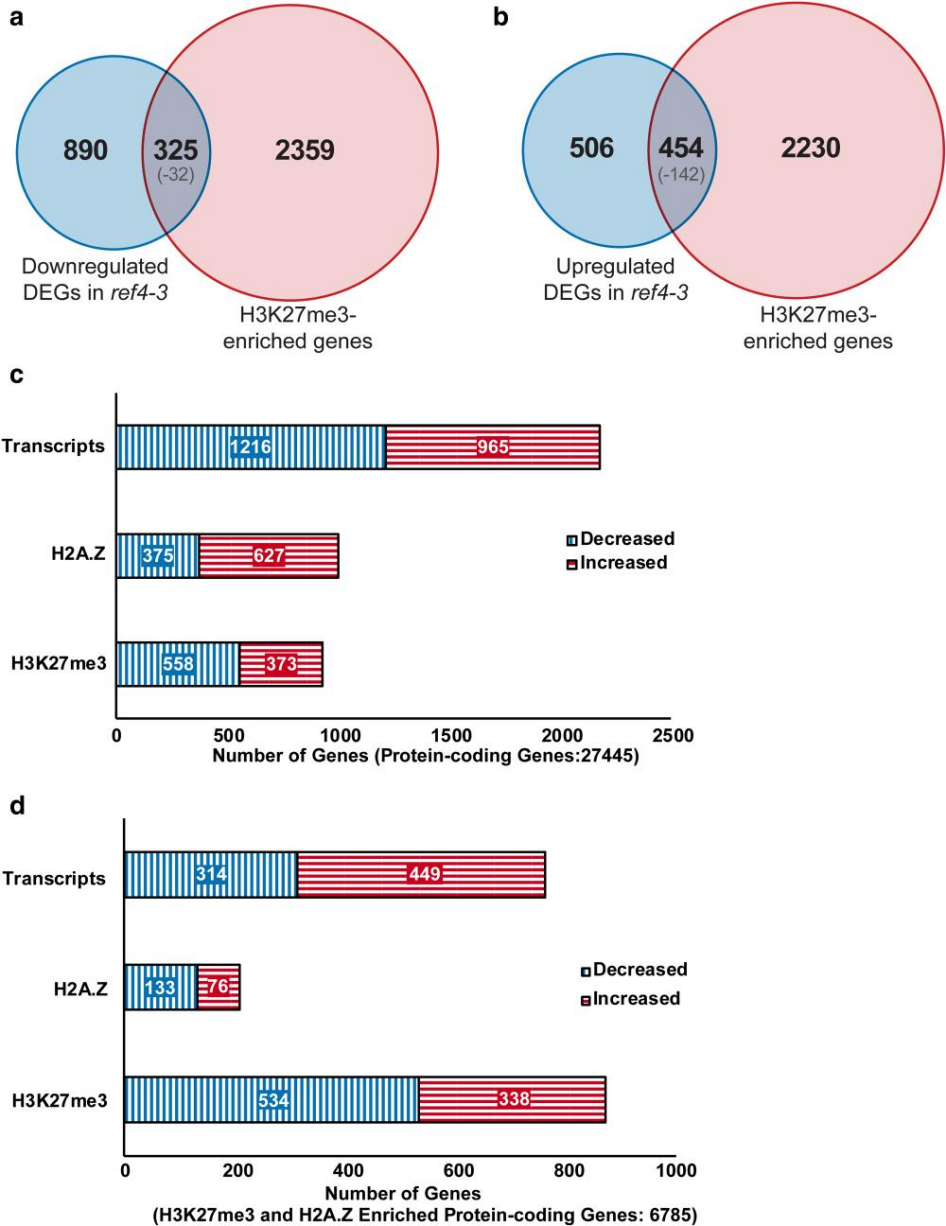
Analysis of genes that are differentially expressed or differentially enriched for H2A.Z or H3K27me3 in *ref4-3* plants. Venn diagrams indicating the intersection between genes identified in our RNA-seq analysis as exhibiting decreased a) or increased b) transcript levels in *ref4-3* relative to wild-type plants and genes identified by our ChIP-seq analysis as H3K27me3 enriched. The log_10_(*P*-value) for each intersection analysis is indicated in the parentheses. Fisher's exact test was used for the statistical analysis. Total number of differentially expressed or differentially enriched genes for H2A.Z or H3K27me3 in *ref4-3* plants are indicated c) out of the set of TAIR10 annotated protein-coding genes and d) out of the set of TAIR10 annotated protein-coding genes that are marked by both H3K27me3 and H2A.Z in either wild-type or *ref4-3* plants. Transcripts, genes that are differentially expressed in RNA-seq analysis in *ref4-3* relative to wild-type plants. H2A.Z, genes that are differentially enriched for H2A.Z in ChIP-seq analysis. H3K27me3, genes that are differentially enriched for H3K27me3 in ChIP-seq analysis. The total number of genes queried in each analysis is indicated in the parentheses.

Our integrated analysis revealed not only a sizable perturbation of the transcriptome, as has been previously described (Dolan *et al*. 2017; Mao *et al*. 2019a), but also that chromatin-based features likely contribute to that perturbation. We observed that genes enriched for H3K27me3 are overrepresented in genes exhibiting altered transcript levels in *ref4-3* plants (Fig. 2a and b), which is consistent with the relationship we observed using previously published data (Supplementary Fig. 1). Our RNA-seq analysis of wild-type and *ref4-3* plants revealed a total of 2,181 DEGs (Fig. 2c; Supplementary Tables 6 and 7). In addition, we identified a total of 1,002 genes that are differentially enriched for H2A.Z and 931 genes that are differentially enriched for H3K27me3 in *ref4-3* plants relative to wild-type plants in the ChIP-seq analysis (Fig. 2c; Supplementary Tables 8–11) demonstrating that *ref4-3* has a sizeable impact on these chromatin features as suggested by our metagene plots (Fig. 1). Consistent with these plots, more protein-coding genes exhibit decreased H3K27me3 levels than increased (Fig. 2c, 558 vs 373) (Supplementary Tables 8 and 9). In contrast, we identified more protein-coding genes with increased H2A.Z than decreased (Fig. 2c, 627 vs 375) (Supplementary Tables 10 and 11) when examining all protein-encoding genes with a cutoff of 1.2-fold change.

Metagene plots reflect average enrichment of a feature at a given set of loci. As averages, they do not reveal the absolute number of genes exhibiting an increase or a decrease or the magnitude of the change at a specific locus, nor do they uncover possible discrete populations of genes exhibiting distinct patterns of enrichment. We therefore constrained our analysis of differential enrichment of H3K27me3 and H2A.Z by examining only those protein-encoding genes enriched for both H3K27me3 and H2A.Z, thus using the same set of genes that we did for Fig. 1c and d. Using this set of genes, we observed a relationship that was consistent with that depicted in those metagene plots: a greater number of genes exhibit decreased H3K27me3 enrichment than increased and, similarly, a greater number of genes exhibit decreased H2A.Z enrichment than increased (Fig. 2d). Nonetheless, it is evident that *ref4-3* results in a range of impacts on these chromatin features at protein-encoding genes.

**Fig. 3.**
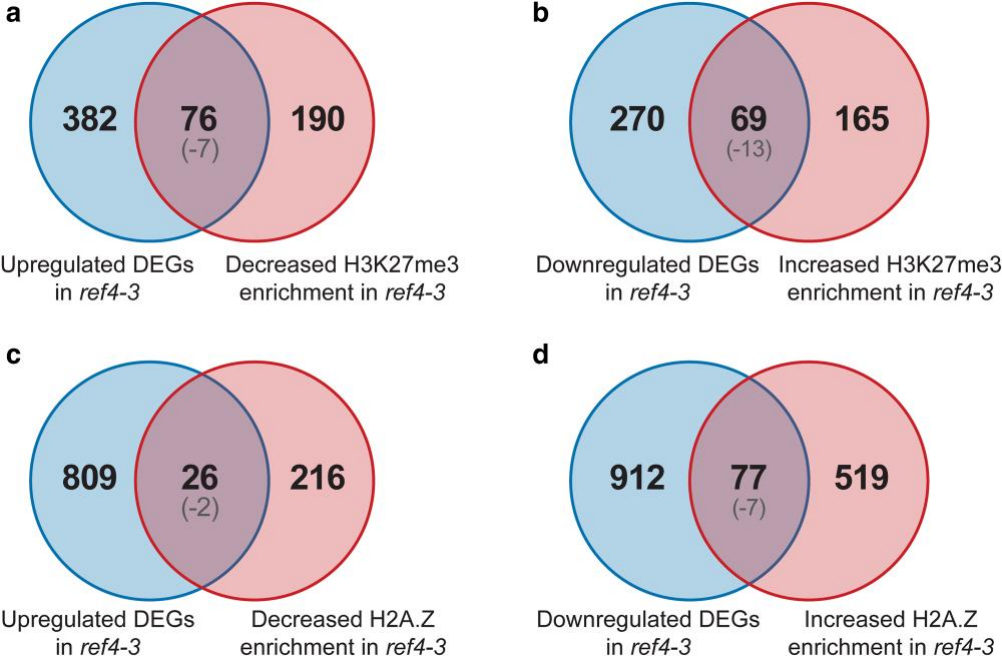
Intersection analyses of genes that exhibit differential expression and/or differential enrichment of H3K27me3 or H2A.Z in *ref4-3* plants. Venn diagrams indicating the intersection between protein-coding genes exhibiting a) increased transcript levels and decreased H3K27me3 enrichment, b) decreased transcript levels and increased H3K27me3 enrichment, c) increased transcript levels and decreased H2A.Z enrichment, and d) decreased transcript levels and increased H2A.Z enrichment in *ref4-3* relative to wild-type plants. Only genes that were expressed and enriched for the mark of interest (H3K27me3 or H2A.Z) in wild-type or *ref4-3* plants were included in this analysis. The log_10_(*P*-value) for each intersection analysis is indicated in the parentheses. Fisher's exact test was used for the statistical analysis.

We examined our RNA-seq data to determine the extent to which the observed perturbation of H3K27me3/H2A.Z (Fig. 1) predicted the transcript phenotype of *ref4-3* plants. In total, 35% of DEGs are enriched for H3K27me3 and H2A.Z (763 of 2,181 genes) whereas only 25% of total protein-coding genes are marked by both H3K27me3 and H2A.Z (6,785 of 27,445 genes) (Fig. 2d). To further examine the relationship between H3K27me3 and H2A.Z enrichment and gene expression, we undertook intersection analyses of genes that are differentially expressed and genes that are differentially enriched for H3K27me3 and/or H2A.Z in *ref4-3* plants relative to wild-type plants (Fig. 3). We observed a modest overrepresentation of genes that exhibit decreased H3K27me3 and/or H2A.Z with genes that exhibit increased transcript levels. Similarly, there was modest overrepresentation of genes that exhibit increased levels of these marks with genes that exhibit decreased transcript levels. Further, scatter plot analysis of log_2_ fold change in transcript levels vs log_2_ fold change of H3K27me3 (Supplementary Fig. 3a) or of H2A.Z (Supplementary Fig. 3b) in *ref4-3* vs wild-type plants reveals a modest negative correlation between changes in H2A.Z or H3K27me3 and transcript levels. These analyses suggest that a limited portion of the transcript phenotype observed in *ref4-3* plants is likely to be directly due to altered levels of H3K27me3 and/or H2A.Z.

**Fig. 4.**
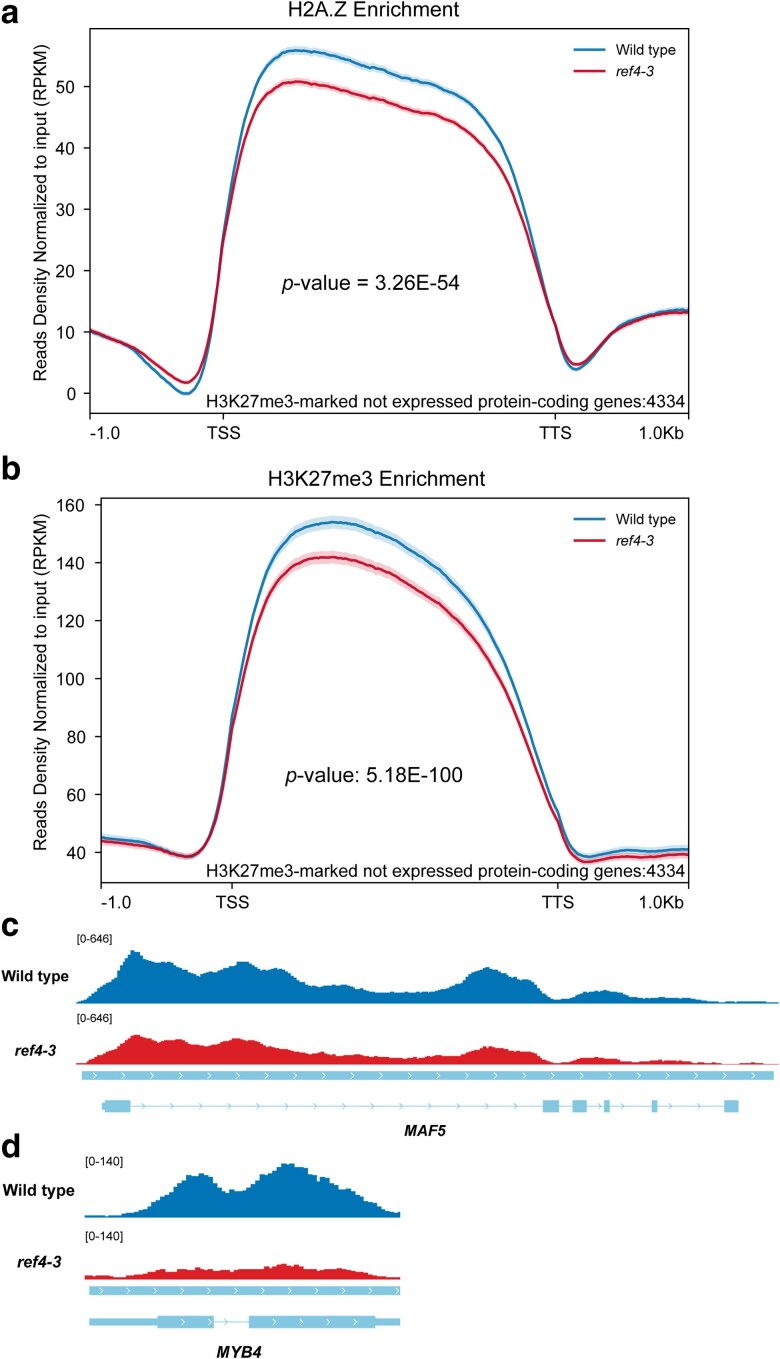
H3K27me3 and H2A.Z enrichment is reduced in *ref4-3* plants at nonexpressed loci. Metagene profiles were used to examine the impact of *ref4-3* on a) average H3K27me3 enrichment level and b) average H2A.Z enrichment level at nonexpressed protein-coding genes marked with H3K27me3. Representative ChIP-seq genome browser views of H3K27me3 enrichment depict c) *MAF5*, a gene identified as H3K27me3-enriched that exhibits decreased H3K27me3 and increased transcript levels in *ref4-3*, and d) *MYB4*, a gene that is not identified as H3K27me3-enriched but also exhibits decreased H3K27me3 levels and increased transcript levels in *ref4-3*. Enrichment for H3K27me3 is normalized to the input for c) and d). The range of RPKM values in c) and d) after autoscaling is indicated in the top left corner. In the metagene profiles, RPKM are generated as described in “Materials and methods” and are representative of merged 2 independent biological replicates of each genotype in which the IP sample of the mark of interest is normalized to its corresponding input DNA. RPKM values are plotted with respect to gene regions that are scaled to 2,500 bp on the *x*-axes. The total number of genes that are included in each plot is indicated at the lower right corner. Data are representative of merged 2 independent biological replicates for either 21-day-old wild-type or *ref4-3* plants. The standard error associated with each analyzed genomic region depicted on the trend line is indicated by the shading surrounding each line. In addition, the significance of the difference in the average level of chromatin-associated features in wild-type and *ref4-3* plants was determined by a paired sample *t*-test and is indicated on each plot. TSS, transcription start site; TTS, transcription termination site.

It is well known that H3K27me3 enrichment at a given locus is anticorrelated with expression of that locus (Zhang *et al*. 2015b; Wiles and Selker 2017; Shen *et al*. 2021). The preceding analyses were thus consistent with an indirect effect of *ref4-3* on H3K27me3 enrichment that aligns with the canonical role of Mediator in gene expression: a mutation of a Mediator tail subunit would be predicted to alter expression of affected loci, leading to altered H3K27me3 enrichment as an indirect effect of altered gene expression. We explicitly analyzed the impact of *ref4-3* on enrichment of H3K27me3 and H2A.Z at H3K27me3-enriched loci that are not detectably expressed to test the hypothesis that altered H3K27me3 enrichment at a gene in *ref4-3* plants is not dependent on altered expression of that gene (Fig. 4a and b). The resulting metagene plots clearly demonstrate an overall reduction of both H3K27me3 and H2A.Z at H3K27me3-marked loci that are not expressed at a detectable level. The corresponding heatmaps again indicate that these trends are associated with similar effects on most genes (Supplementary Fig. 4). Importantly, expression of genes coding for factors previously linked to H3K27me3 homeostasis such as PRC2 and histone H3K27 demethylases is not altered in *ref4-3* plants (Supplementary Fig. 5). These findings indicate that *ref4-3* (most likely in the context of the Mediator complex) can act in a transcription-independent fashion to alter H3K27me3 and H2A.Z at H3K27me3-enriched loci.

The altered distribution of H2A.Z and H3K27me3 in *ref4-3* plants presents a novel context in which to examine the relationship between these modifications. Analysis of the intersection between genes exhibiting altered H3K27me3 and/or H2A.Z in *ref4-3* plants (Tables 1–3) reveals a robust positive correlation between altered enrichment of these features regardless of the expression status of the locus (above or below the limit of detection as determined by RNA-seq). Based on the consistency of this correlation, we propose *ref4-3* preferentially impacts a chromatin state that is defined in part by both H3K27me3 and H2A.Z. Chromatin states defined in part by the presence of these features have been previously identified (Sequeira-Mendes *et al*. 2014; Jamge *et al*. 2023). The positive correlation is also consistent with the proposed role for H2A.Z in deposition of H3K27me3 (Carter *et al*. 2018).

**Table 1.** Intersection analyses of protein-coding genes that are differentially expressed and/or differentially enriched for H3K27me3 or H2A.Z.

Gene set A	Number of set A	Gene set B	Number of set B	Predicted	Observed	Percentage of set A	Percentage of set B	*P*-value	Significance
Increased H2A.Z levels	76	Increased H3K27me3 levels	338	3.79	28	37	8	6.34E^−18^	***
Increased H2A.Z levels	76	Decreased H3K27me3 levels	534	5.98	6	8	1	0.56	
Decreased H2A.Z levels	133	Increased H3K27me3 levels	338	6.63	6	5	2	0.66	
Decreased H2A.Z levels	133	Decreased H3K27me3 levels	534	10.47	40	30	7	2.69E^−14^	**
Increased transcript levels	458	Increased H3K27me3 levels	234	38.87	9	2	4	1.00	
Increased transcript levels	458	Decreased H3K27me3 levels	266	44.19	76	17	29	1.76E^−07^	*
Decreased transcript levels	339	Increased H3K27me3 levels	234	28.77	69	20	29	1.06E^−13^	**
Decreased transcript levels	339	Decreased H3K27me3 levels	266	32.71	11	3	4	1.00	
Increased transcript levels	835	Increased H2A.Z levels	596	36.06	14	2	2	1.00	
Increased transcript levels	835	Decreased H2A.Z levels	242	14.64	26	3	11	3.18E^−03^	*
Decreased transcript levels	989	Increased H2A.Z levels	596	42.71	77	8	13	2.58E^−07^	*
Decreased transcript levels	989	Decreased H2A.Z levels	242	17.34	29	3	12	4.40E^−03^	*

Genes exhibiting detectable transcript levels in RNA-seq and enriched for the relevant mark were included in each intersection analysis.

Observed, number of genes in common between the indicated gene sets; Predicted, number of genes predicted to be in common by chance; Percentage of gene set A, percentage of genes in common in the gene set A; Percentage of gene set B, percentage of genes in common in the gene set B; *P-*value, obtained using Fisher's exact test with a null hypothesis of an intersection that is no greater than expected by chance.

Significance: *α ≤ 0.05, **α ≤ 10^−10^, and ***α ≤ 10^−15^.

**Table 2.** Intersection analyses of differentially enriched protein-coding genes that are expressed.

Gene set A	Number of set A	Gene set B	Number of set B	Predicted	Observed	Percentage of set A	Percentage of set B	*P-*value	Significance
Increased H2A.Z levels	56	Increased H3K27me3 levels	222	4.65	22	39	10%	1.02E^−10^	*
Increased H2A.Z levels	56	Decreased H3K27me3 levels	261	5.46	5	9%	2%	0.65	
Decreased H2A.Z levels	58	Increased H3K27me3 levels	222	4.81	6	10%	3%	0.35	
Decreased H2A.Z levels	58	Decreased H3K27me3 levels	261	5.66	20	34%	8%	1.82E^−07^	*

Protein-coding genes exhibiting detectable transcript levels in RNA-seq and enriched for both H3K27me3 and H2A.Z were included in this analysis. Observed: number of genes in common between the indicated gene sets. Predicted: number of genes predicted to be in common by chance. Percentage of gene set A: percentage of genes in common in the gene set A. Percentage of gene set B: percentage of genes in common in the gene set B. *P-*value: obtained using Fisher's exact test with a null hypothesis of an intersection that is no greater than expected by chance. Significance: *α ≤ 0.05.

**Table 3.** Intersection analyses of differentially enriched protein-coding genes that are not expressed.

Gene set A	Number of set A	Gene set B	Number of set B	Predicted	Observed	Percentage of set A	Percentage of set B	*P-*value	Significance
Increased H2A.Z levels	20	Increased H3K27me3 levels	116	0.56	6	30	5	1.25E^−05^	*
Increased H2A.Z levels	20	Decreased H3K27me3 levels	273	1.33	1	5	0	0.75	
Decreased H2A.Z levels	75	Increased H3K27me3 levels	116	2.12	0	0	0	1.00	
Decreased H2A.Z levels	75	Decreased H3K27me3 levels	273	4.98	20	27	7	4.07E^−08^	*

Protein-coding genes exhibiting undetectable transcript levels in RNA-seq and enriched for both H3K27me3 and H2A.Z were included in this analysis.

Observed, number of genes in common between the indicated gene sets; Predicted, number of genes predicted to be in common by chance; Percentage of gene set A, percentage of genes in common in the gene set A; Percentage of gene set B, percentage of genes in common in the gene set B; *P-*value, obtained using Fisher's exact test with a null hypothesis of an intersection that is no greater than expected by chance.

Significance: *α ≤ 0.05.

### Previously characterized metabolic and development phenotypes of *ref4-3* may result from a chromatin-based mechanism

Examination of expression of genes associated with the phenylpropanoid biosynthetic pathway and of genes characterized as regulators of phenylpropanoid and secondary cell wall biosynthesis revealed an impact of *ref4-3* similar to that previously characterized (Supplementary Tables 12 and 13) (Dolan *et al*. 2017; Mao *et al*. 2019a). In particular, we observed genes associated with phenylpropanoid biosynthesis generally exhibited decreased transcript levels in *ref4-3* plants, including genes associated with early steps in the phenylpropanoid pathway such as *PAL* (PHE ammonia lyase), *C4H* (cinnamate-4-hydroxylase), *4CL* (4-coumarate:CoA ligase), *HCT* (hydroxycinnamoyl-CoA shikimate/quinate hydroxycinnamoyl transferase), and *C3'H* (coumarate 3-hydroxylase) and genes that contribute to transcriptional and posttranslational regulation, such as *MYB4* (Supplementary Tables 12 and 13). Similarly, we observed that genes associated with flavonoid biosynthesis or transcriptional regulation are generally increased in *ref4-3* plants as observed previously (Supplementary Table 14).

A gene ontology (GO) analysis of genes exhibiting altered enrichment of H3K27me3 did not reveal an enrichment in genes associated with the phenylpropanoid or flavonoid biosynthesis (Supplementary Table 15). This finding was not unanticipated in light of the substantial portion of the transcript phenotype that is not associated with altered H3K27me3 enrichment (Fig. 3). Nevertheless, we undertook a candidate gene approach to explore the possible contribution of altered H3K27me3 enrichment to previously characterized *ref4-3* transcript and developmental phenotypes. The candidates included *MYB4*, a transcriptional regulator that plays a broad role in the regulation of genes associated with flavonoid biosynthesis (Jin *et al*. 2000; Wang *et al*. 2020) and that exhibits elevated expression in *ref4-3* plants as previously observed (Supplementary Table 13).

Although *MYB4* was not identified as an H3K27me3-enriched locus by our bioinformatic pipeline due to the stringent selection criteria (see “Materials and methods”), visual inspection of H3K27me3 enrichment at *MYB4* in wild-type and *ref4-3* plants clearly reveals a decrease of H3K27me3 that is comparable to that observed at *MAF5*, which was identified as a locus exhibiting differential enrichment of H3K27me3 by our analysis (Fig. 4c and d). High signal thresholds for calling a locus enriched for a chromatin feature result in robust reproducible signals but can result in false negatives (Nakato and Sakata 2021; Eder and Grebien 2022). Importantly, mutation of *MYB4* in *ref4-3* plants contributes to increased expression of genes associated with phenylpropanoid biosynthesis, increased levels of anthocyanin and proanthocyanidin, and an amelioration of developmental phenotypes observed in *ref4-3* plants (Wang *et al*. 2020). Further, the transcript level of *MYB4* is increased 2.8-fold in *clf swn* plants that lack H3K27 histone methyltransferases that act in the shoot to generate H3K27me3 (Shu *et al*. 2019). Combined, these observations are consistent with a model in which elevated expression of *MYB4* in *ref4-3* plants and the ensuing phenotypic consequences result from reduced H3K27me3 at *MYB4*, a mechanism of action that is consistent with the impact of *ref4-3* on H3K27me3 enrichment at numerous other loci (Fig. 2).

### *ref4-3* impairs expression of MYC2-dependent genes

**Fig. 5.**
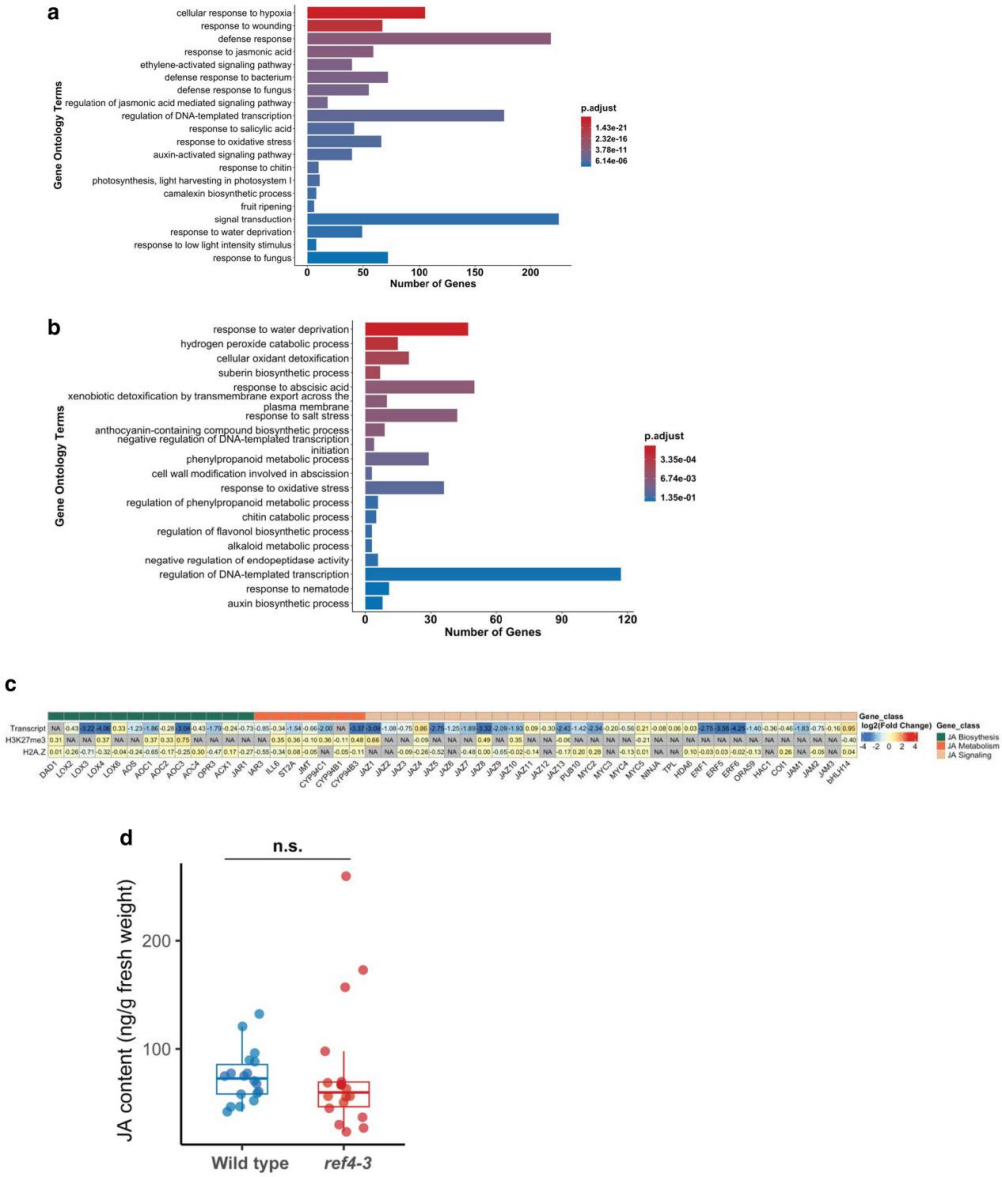
Expression of genes associated with JA biosynthesis and response are misregulated in *ref4-3* plants. a) GO enrichment analysis of genes that exhibit decreased transcript levels in *ref4-3* plants. b) GO enrichment analysis of genes that exhibit increased transcript levels in *ref4-3* plants. c) Relative expression and enrichment of H3K27me3 and H2A.Z of JA-associated genes in *ref4-3* plants. d) JA abundance in 3-week-old shoot tissue (*n* = 18) in different genetic backgrounds. JA content was determined by calculating the relative peak area ratio to the known amount of internal standard (JA-d_5_) added before metabolite extraction and then normalized to the fresh weight of tissue. Quantification was repeated twice, and the representative data are shown here. The significance of the difference in JA levels between wild type and *ref4-3* was determined by Welch's *t-*test (2-tail). n.s. denotes not significant.

GO term enrichment analysis of DEGs in *ref4-3* revealed overrepresentation of pathways in *ref4-3* plants similar to those identified previously, including pathways associated with phenylpropanoid metabolic processes, response to salicylic acid, and flavonoid biosynthesis (Fig. 5a and b; Supplementary Table 16). It is worth highlighting that the enrichment in genes associated with flavonoid biosynthesis is consistent with our observed transcript (Supplementary Table 13) and H3K27me3 enrichment phenotype (Fig. 4d) for *MYB4*. We also observed a striking overrepresentation of downregulated genes associated with JA response and regulation by JA as well as JA-associated processes such as defense response and wounding (Wasternack and Song 2017; Hewedy *et al*. 2023).

In light of this distinctive transcriptome phenotype, we examined the overlap of the genes that we identified as differentially expressed in *ref4-3* plants with another set of genes that were previously identified as differentially expressed in *ref4-3* plants (Dolan *et al*. 2017) (Supplementary Fig. 6a and b). Although this analysis revealed significant overlap, 62% of genes (419 of 681) that we identified as exhibiting increased expression in *ref4-3* plants were not identified previously (Supplementary Fig. 6a). Additionally, 82% of genes (852 of 1,037) that we identified as exhibiting decreased expression were also not identified previously (Supplementary Fig. 6b). To further investigate the basis of our transcript phenotype, we undertook multidimensional scaling (MDS) analysis to compare our data to 2 previous RNA-seq analyses of *ref4-3* plants which did not exhibit such a dramatic perturbation of the JA-associated transcriptome (Dolan *et al*. 2017; Mao *et al*. 2019a). This comparison revealed that the 3 data sets clustered by experimental condition rather than by genotype (Supplementary Fig. 6c). MDS analysis of these 3 RNA-seq analyses thus indicates that the growth condition of a given experiment has a substantial impact on the wild-type transcriptome, which then represents a distinct “ground state” for examining the effect of *ref4-3* on transcript levels (and possibly chromatin states as well). Although plants in all 3 analyses were grown under a typical long-day regimen of a 16-h light/8-h dark photoperiod, they were harvested at slightly different ages and were grown in different chambers using different light sources and under different light intensities (Supplementary Fig. 6c). Our MDS analysis strongly suggests that one or more of these experimental variables can have a substantial impact on the transcriptome of wild-type plants, which in turn likely alters the impact of a genetic perturbation on that transcriptome.

Our analysis of *ref4-3* plants under our growth conditions revealed a wide-ranging alteration of JA-associated gene expression that is not strongly associated with a particular change in chromatin state in *ref4-3* plants, including decreased transcript levels for genes that play critical roles in JA biosynthesis, regulation of JA signaling, and response to JA in *ref4-3* plants (Fig. 5c; Supplementary Table 17). We analyzed JA content in whole shoot tissue of wild-type and *ref4-3* plants by LS-MS to test the hypothesis that this transcript phenotype reflected altered JA homeostasis in *ref4-3* plants. We observed that *ref4-3* plants maintain JA levels that are indistinguishable from wild-type plants (Fig. 5d), suggesting that another process contributed to altered expression of these loci.

**Fig. 6.**
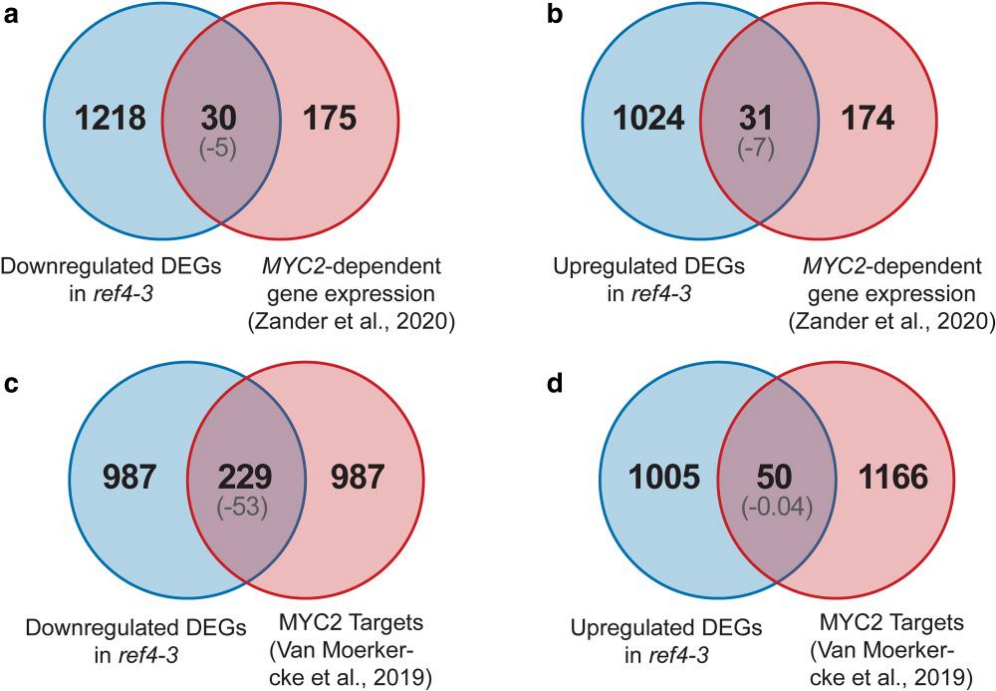
Direct targets of MYC2 are greatly overrepresented among downregulated genes in *ref4-3* plants. Venn diagrams indicating the intersection between genes that are a) downregulated in *ref4-3* and exhibit altered expression in *myc2* plants (Zander *et al*. 2020); b) upregulated in ref4-3 and exhibit altered expression in *myc2* plants (Zander *et al*. 2020); c) downregulated in *ref4-3* and direct targets of MYC2 (Van Moerkercke *et al*. 2019); and d) upregulated in *ref4-3* and direct targets of MYC2 (Van Moerkercke *et al*. 2019). The log_10_(*P*-value) for each intersection analysis is indicated in the parentheses. Fisher's exact test was used for the statistical analysis.

MYC2 plays a key role in transcriptional regulation of a variety of processes in plants, including those associated with JA homeostasis (Wang *et al*. 2017; Van Moerkercke *et al*. 2019; Zhang *et al*. 2020). Intriguingly, we observed that transcript levels of *MYC2* are decreased 5-fold in *ref4-3* plants, suggesting that reduced expression of *MYC2* contributed to the transcript phenotype (Fig. 5c). We observed a relatively small overlap, however, between genes that are misregulated in *ref4-3* plants and genes that were previously identified as exhibiting altered transcript levels in *myc2* plants (Zander *et al*. 2020) (Fig. 6a and b). Loss of *MYC2* has previously been shown to result in a modest transcript phenotype, which likely reflects functional overlap with *MYC3* and *MYC4*, which code for related transcription factors that act in common transcriptional circuits with *MYC2* (Fernandez-Calvo *et al*. 2011; Wang *et al*. 2017; Zander *et al*. 2020; Zhang *et al*. 2020). To test the hypothesis that *ref4-3* specifically alters expression of genes that are direct targets of MYC2 independently of a requirement for *MYC2*-dependent expression, we examined the intersection between genes that are misregulated in *ref4-3* and genes that were previously characterized by ChIP-seq as direct targets of MYC2 (Van Moerkercke *et al*. 2019). We observed that *ref4-3* impaired expression of a substantial subset of these genes (229 of 1,216 downregulated genes, *P*-value < 10^−53^) (Fig. 6c and d), consistent with the hypothesis that the presence of a MYC2-binding site conferred *ref4-3*-associated transcriptional inhibition at a number of loci.

### *MED25* is necessary for *ref4-3*-dependent inhibition of expression of targets of MYC2

**Fig. 7.**
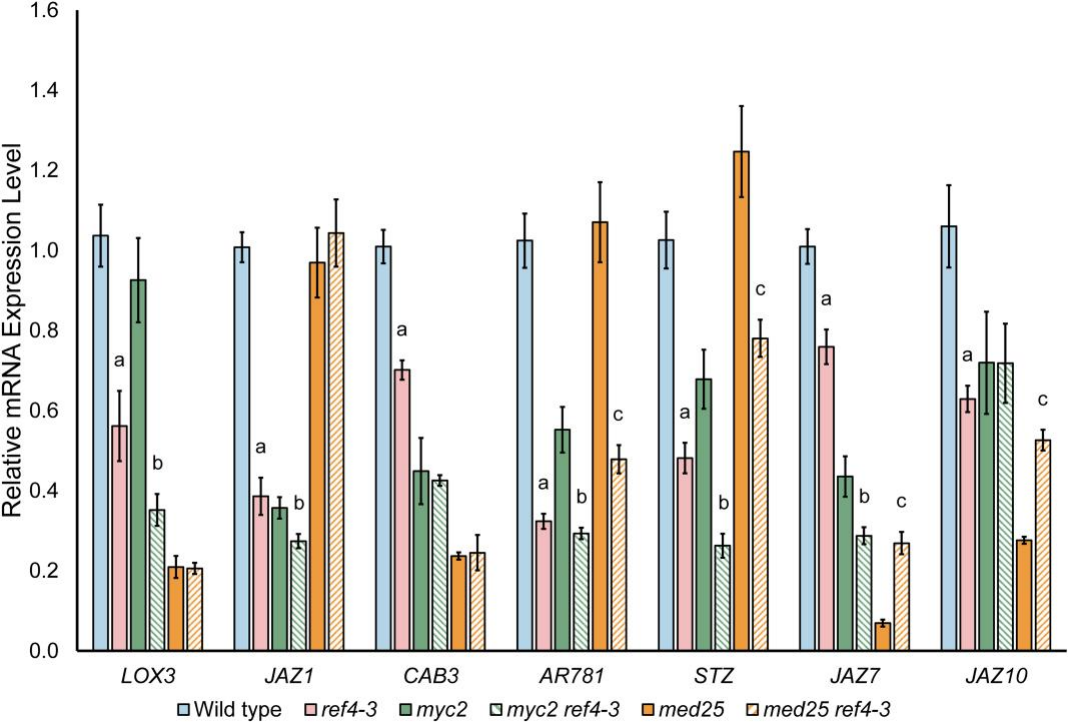
Mutation of *MED25* can alleviate inhibition of MYC2-targeted genes in *ref4-3* plants. RT-qPCR analysis was used to examine the impact of *ref4-3* on the relative expression of MYC2 target genes in the absence of *MYC2* or *MED25*. Transcript levels are normalized to the expression in wild-type plants, and 18S was used as a reference gene. Data points are the average of 3 biological replicates. The statistically significant level of gene expression was determined with Student's *t*-test (2-tail). Letters indicate the specific comparison exhibits a *P* < 0.05 at the examined locus. a, *ref4-3* plants relative to wild-type plants; b, *myc2 ref4-3* plants relative to *myc2* plants; c, *med25 ref4-3* plants relative to *med25* plants.

Our data suggested that *ref4-3* preferentially acts to impair expression of some Myc2 protein (MYC2) targets in a *MYC2*-dependent fashion via MYC2-dependent recruitment of Mediator containing the altered MED5b subunit. To test this hypothesis, we investigated whether the loss of *MYC2* in *ref4-3* plants suppressed the transcript phenotype of *MYC2* targets by using RT-qPCR to examine transcript levels in wild-type, *ref4-3*, *myc2*, and *ref4-3 myc2* plants. For this analysis, we selected a panel of 7 genes that exhibited decreased expression in *ref4-3* plants and were direct targets of MYC2 (Van Moerkercke *et al*. 2019). All 7 selected genes exhibited reduced transcript levels in *ref4-3* relative to wild-type plants in the RT-qPCR analysis, in agreement with our RNA-seq analysis (Fig. 7). Loss of *MYC2* had a modest impact on reduced transcript levels associated with *ref4-3*: only 2 genes (*CAB3* and *JAZ10*) exhibited no detectable decrease in expression in *myc2 ref4-3* vs *myc2* plants, whereas *ref4-3* still impaired expression of the other 5 genes in *myc2* plants. These data thus indicate that *MYC2* is not necessary for *ref4-3* to impair expression of direct targets of MYC2.

As noted above, MYC2 acts in common transcriptional circuits with MYC3 and MYC4 (Fernandez-Calvo *et al*. 2011; Gao *et al*. 2016). MYC2 can form heterodimers with MYC3 and MYC4, and MYC3 exhibits DNA binding specificity that is indistinguishable from MYC2 in vitro (Fernandez-Calvo *et al*. 2011). RNA-seq analysis of *myc2* plants has previously revealed a relatively modest transcript phenotype relative to the number of MYC2 binding sites in the absence of JA treatment (Zander *et al*. 2020). Analysis of the transcript levels of our panel of selected genes similarly reveals a similarly modest impact on transcript levels associated with loss of *MYC2* as 5 out of 7 selected genes exhibit less than a 2-fold reduction in transcript levels in *myc2* plants (Fig. 7, *myc2* vs wild type). Critically, MYC2, MYC3, and MYC4 all interact with MED25 (Cevik *et al*. 2012; Zhang *et al*. 2015a; Takaoka *et al*. 2022), and this interaction is considered to be a key mechanism by which Mediator is recruited to MYC2/3/4-dependent loci.

Given the functional redundancy of MYC2/3/4 and the common interaction of these transcription factors with MED25, we examined whether loss of *MED25* could abrogate the MYC2-associated transcript phenotype of *ref4-3*. We examined the transcript level of the same panel of genes in *med25* and *ref4-3 med25* lines. We observed that in 5 out of the 7 genes, *ref4-3* no longer impaired expression in the context of *med25*. Three of the genes (*LOX3*, *JAZ1*, and *CAB3*) exhibit indistinguishable transcript levels in *med25 ref4-3* relative to *med25* whereas 2 of the genes (*JAZ7* and *JAZ10*) exhibit increased transcript levels in *med25 ref4-3* relative to *med25*. These data are consistent with a mechanism of action in which *ref4-3* preferentially impairs expression at some loci to which Mediator is recruited via the interaction between MYC transcription factors and MED25.

## Discussion

Previous characterization of *ref4-3*, a semidominant point mutation of the tail module of Mediator (Bonawitz *et al*. 2012), revealed its impact on gene expression associated with metabolism and the contribution of other Mediator tail subunits to that perturbation (Stout *et al*. 2008; Bonawitz *et al*. 2012; Dolan *et al*. 2017; Wang *et al*. 2020). Here we show that *ref4-3* can also impair expression of genes that are direct targets of MYC2, a key transcriptional regulator involved in JA homeostasis and other processes (Wang *et al*. 2017; Van Moerkercke *et al*. 2019; Zhang *et al*. 2020), and that genetic ablation of MED25, a tail subunit previously demonstrated to interact directly with MYC2 and related transcription factors (Cevik *et al*. 2012; Zhang *et al*. 2015a; Takaoka *et al*. 2022), can ameliorate this impairment. We also make the surprising observation that *ref4-3* leads to a modest reduction in H3K27me3 at H3K27me3-enriched loci, which is accompanied by a reduction of H2A.Z at these loci. This reduction of H3K27me3 is observed at loci that are not transcribed at detectable levels, suggesting that Mediator carrying a point mutation in Med5b contributes to H3K27me3 homeostasis via a transcription-independent mechanism. Our studies highlight the diverse effects of a point mutation in a tail subunit of plant Mediator on transcriptional regulation and raise the possibility that Mediator plays a role in the distinct H3K27me3 landscape of plants.

**Fig. 8.**
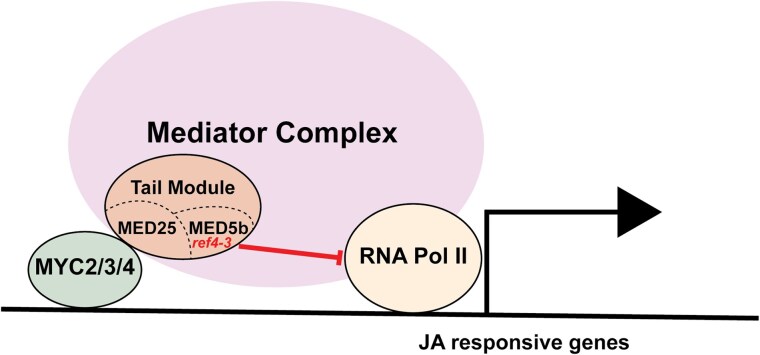
Model of recruitment of Mediator complex that contains mutant version of MED5b corresponding to *ref4-3* to MYC2-dependent loci. Upon recruitment of Mediator via the interaction between MYC2 (or the related transcription factors MYC3 and MYC4), the semidominant point mutation of Med5b (*ref4-3*) is placed in a context in which it has the ability to inhibit gene expression (denoted by bar).

RNA-seq of 21-day-old *ref4-3* plants revealed a notable impact on expression of genes directly regulated by MYC2. 18.8% of genes identified as direct targets of MYC2 by ChIP-seq (Van Moerkercke *et al*. 2019) exhibited decreased transcript levels in *ref4-3* plants (Fig. 6c). Previous biochemical characterization of MYC2 indicates that MYC2 and related transcription factors MYC3 and MYC4 can recruit Mediator by directly interacting with the MED25 tail subunit (Cevik *et al*. 2012; Zhang *et al*. 2015a; Takaoka *et al*. 2022). Our genetic data are consistent with this biochemical model of action. Analysis of candidate gene expression by RT-qPCR reveals that 5 of 7 genes that are direct targets of MYC2 no longer exhibit decreased expression in *ref4-3* plants if *MED25* is absent. Thus, our data suggest that Mediator containing a semidominant mutant version of Med5b can impair gene expression when recruited to these loci via a MYC2 binding site—MYC2/3/4–MED25 recruitment pathway (Fig. 8).

In addition to revealing an impact of *ref4-3* on expression of direct targets of MYC2, our analyses emphasize the responsiveness of the transcriptome of wild-type plants to growth conditions. Previous characterization of *ref4-3* by 2 independent RNA-seq analyses called attention to its impact on gene expression associated with metabolism (e.g. phenylpropanoid biosynthesis) (Dolan *et al*. 2017; Mao *et al*. 2019a). MDS analysis of all 3 RNA-seq data sets to gain insight into the distinct effects of *ref4-3* on the transcriptome in the 3 experiments gave rise to the surprising observation that despite using what would typically be considered highly similar growth conditions and developmental stages for all 3 experiments and the use of a mutant with a distinct developmental phenotype, the experiment rather than the genotype primarily accounted for the observed differences in the transcriptomes (Stout *et al*. 2008; Wang *et al*. 2020) (Supplementary Fig. 6c).

Responsive adaptation to environmental conditions is a hallmark of plant growth and development (Sultan 2000; Schneider 2022). Our comparative analysis of independent replicates of RNA-seq data of identical plant lines from common seed stocks indicates that this high degree of responsiveness extends to the level of the transcriptome as well. Given the highly integrative manner by which Mediator contributes to transcriptional control (Allen and Taatjes 2015; Yang *et al*. 2016; Chen *et al*. 2022; Richter *et al*. 2022), it is perhaps not surprising that different transcriptional ground states (as dictated by a given environmental condition) will reveal different perturbations in response to a semidominant mutation in a Mediator tail subunit. Our findings underscore, however, that providing additional information such as the light sources and/or a sample light spectrum in the “Materials and methods” section may aid in reproducibility of transcript-associated phenotypes observed in different labs.

Despite the apparent impact of slightly altered growth conditions on the transcriptome of plants (Supplementary Fig. 6c), our new analyses reproduced the observation that H3K27me3-enriched genes are overrepresented in genes that exhibit altered expression in *ref4-3* plants (Fig. 2a and b; Supplementary Fig. 1). In this study, we used ChIP-seq to explicitly test the hypothesis that H3K27me3 enrichment was altered in *ref4-3* plants. Our data clearly demonstrate that *ref4-3* results in a modest reduction of levels of H3K27me3 at H3K27me3-enriched loci (Fig. 1). Intriguingly, we also observed this reduction at loci that are not detectably expressed (Fig. 4), indicating that Mediator contributes to H3K27me3 enrichment via a mechanism that is not directly coupled to expression of the locus.

Mediator has previously been implicated in determination of chromatin architecture in animals (Richter *et al*. 2022), including protein–protein interactions linking the Mediator kinase module to H3K27me3 enrichment (Fukasawa *et al*. 2015; Postlmayr *et al*. 2020; Dimitrova *et al*. 2022). Plants exhibit a distinct pattern of enrichment of H3K27me3 relative to animals (Grossniklaus and Paro 2014): in plants, areas of H3K27me3 enrichment are typically observed in a locus-specific fashion, whereas in animals, H3K27me3 enrichment tends to occur in broad swaths of the genome. A number of cis-acting elements have been identified that are likely to contribute to locus-specific deposition of H3K27me3 in plants (Bieluszewski *et al*. 2021; Godwin and Farrona 2022). Our data raise the prospect that Mediator also contributes to deposition of H3K27me3 in plants, with the distinct caveat that the semidominant nature of *ref4-3* imbues the resulting Mediator complex with neomorphic properties.

Another distinguishing feature of H3K27me3-enriched loci in plants in relation to animals is that they are also enriched for the histone variant H2A.Z (Sequeira-Mendes *et al*. 2014; Carter *et al*. 2018; Gomez-Zambrano *et al*. 2019), and it has been proposed that H2A.Z contributes to deposition of H3K27me3 (Carter *et al*. 2018). Our analysis of H2A.Z enrichment by ChIP-seq in *ref4-3* plants revealed that changes in H3K27me3 are strongly correlated with changes in H2A.Z at H3K27me3-enriched loci (Figs. 1 and 4 and Tables 1–3). Our results are thus consistent with previous observations that the presence of H3K27me3 and H2A.Z define a particular chromatin state in Arabidopsis (Sequeira-Mendes *et al*. 2014) and indicate that the relative abundance of this state is perturbed by *ref4-3*. Our data do not indicate, however, whether *ref4-3* contributes specifically to H2A.Z and/or H3K27me3 at these loci.

Although *ref4-3* perturbs H3K27me3 homeostasis, this effect is unlikely to be the primary mechanism by which *ref4-3* alters the transcriptome. In our analysis, *ref4-3* has a substantially greater impact on expression of MYC2 direct targets (Fig. 6c) than on expression of genes that exhibit altered enrichment of H3K27me3 (Table 1). Nevertheless, our data also raise the possibility that altered H3K27me3 homeostasis as a result of *ref4-3* contributes to a substantial aspect of the developmental phenotype of *ref4-3* plants. *MYB4* has previously been demonstrated to be required for expression of the developmental and flavonoid phenotype of *ref4-3* plants (Wang *et al*. 2020). Although *MYB4* was not identified by our statistical criteria as an H3K27me3-enriched locus, visual inspection of H3K27me3 levels at *MYB4* in wild-type and *ref4-3* plants clearly reveals a decrease in H3K27me3 enrichment in *ref4-3* plants that could contribute to the observed increased transcript level of *MYB4* (Fig. 4d). This possibility is worth highlighting as it illustrates how a mutant that leads to a general perturbation in some process may give rise to a distinct phenotype not because of the widespread effect generated by the perturbation but because of the effect of that perturbation on a specific locus, analogous to the identification of *SDC* as a genetic suppressor of the developmental phenotype of *drm1 drm2 cmt3* plants (Henderson and Jacobsen 2008). Our data indicate that when examining the contribution of Mediator in plants to transcriptional regulation of a locus of interest (often due to an emergent developmental or biochemical trait), chromatin-based mechanisms should be considered.

## Supplementary Material

jkae301_Supplementary_Data

## Data Availability

RNA-seq and ChIP-seq data from this article were deposited into the Gene Expression Omnibus (Edgar *et al*. 2002). RNA-seq data can be found under accession number GSE275912, and ChIP-seq data can be found under accession number GSE275810. Supplemental material available at G3 online.
