# Supplementary figures and images for: A semidominant point mutation of Mediator tail subunit MED5b in Arabidopsis leads to altered enrichment of H3K27me3 and reduced expression of targets of MYC2
Source: G3 (Bethesda). 2025 Feb 14;15(3):jkae301. doi: 10.1093/g3journal/jkae301 (PMC11917473; doi:10.1093/g3journal/jkae301)

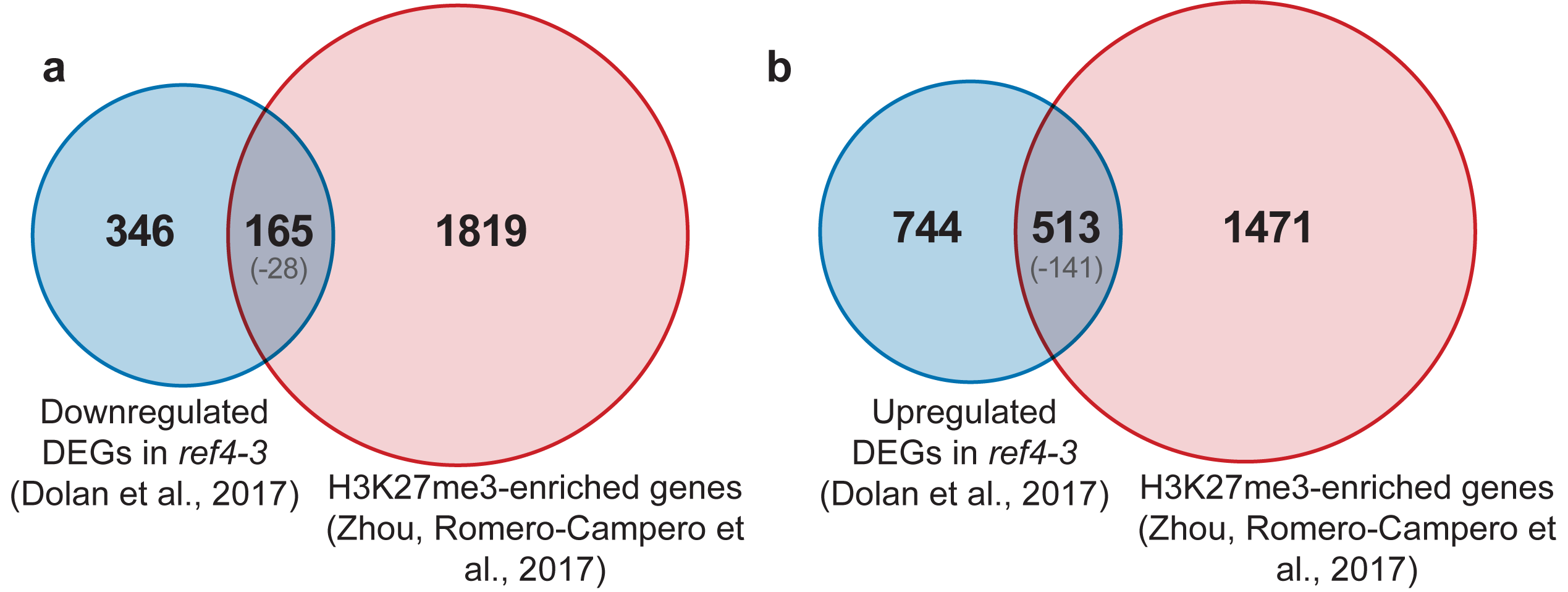

Supplement: jkae301_Supplementary_Data [file jkae301_supplementary_data.zip › Figure_S1_G3-2024-405531.tif]

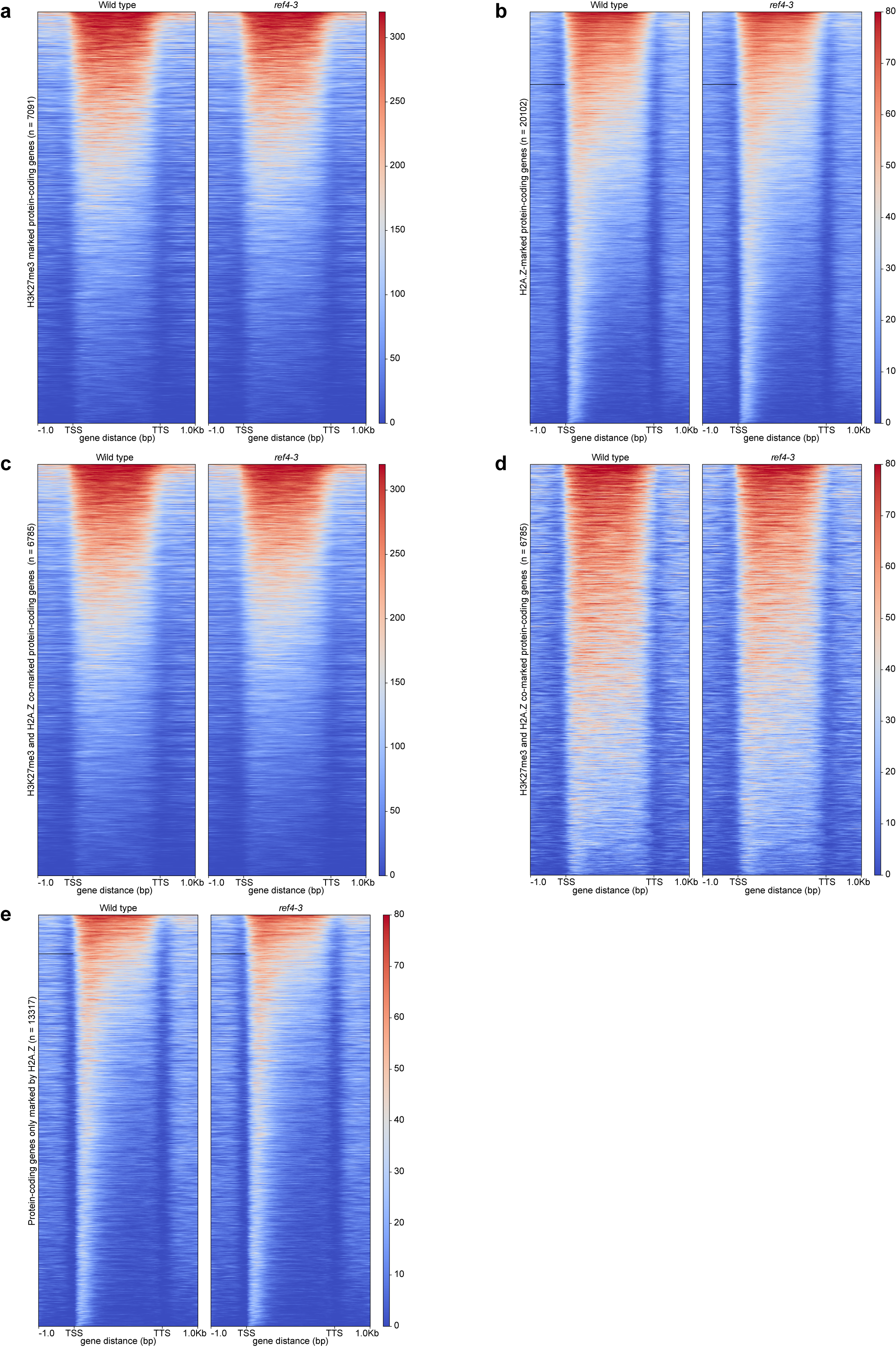

Supplement: jkae301_Supplementary_Data [file jkae301_supplementary_data.zip › Figure_S2_G3-2024-405531.tif]

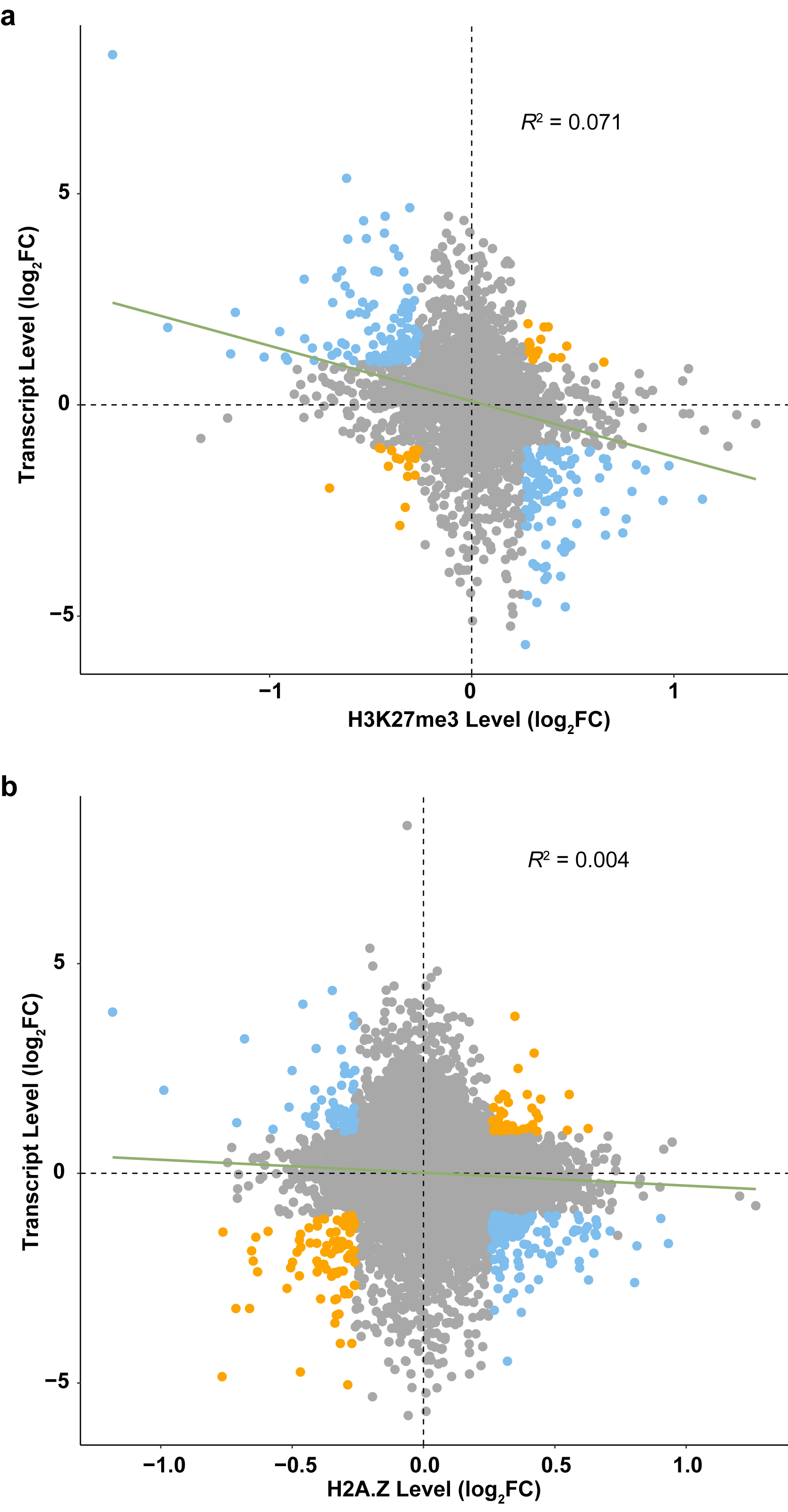

Supplement: jkae301_Supplementary_Data [file jkae301_supplementary_data.zip › Figure_S3_G3-2024-405531.tif]

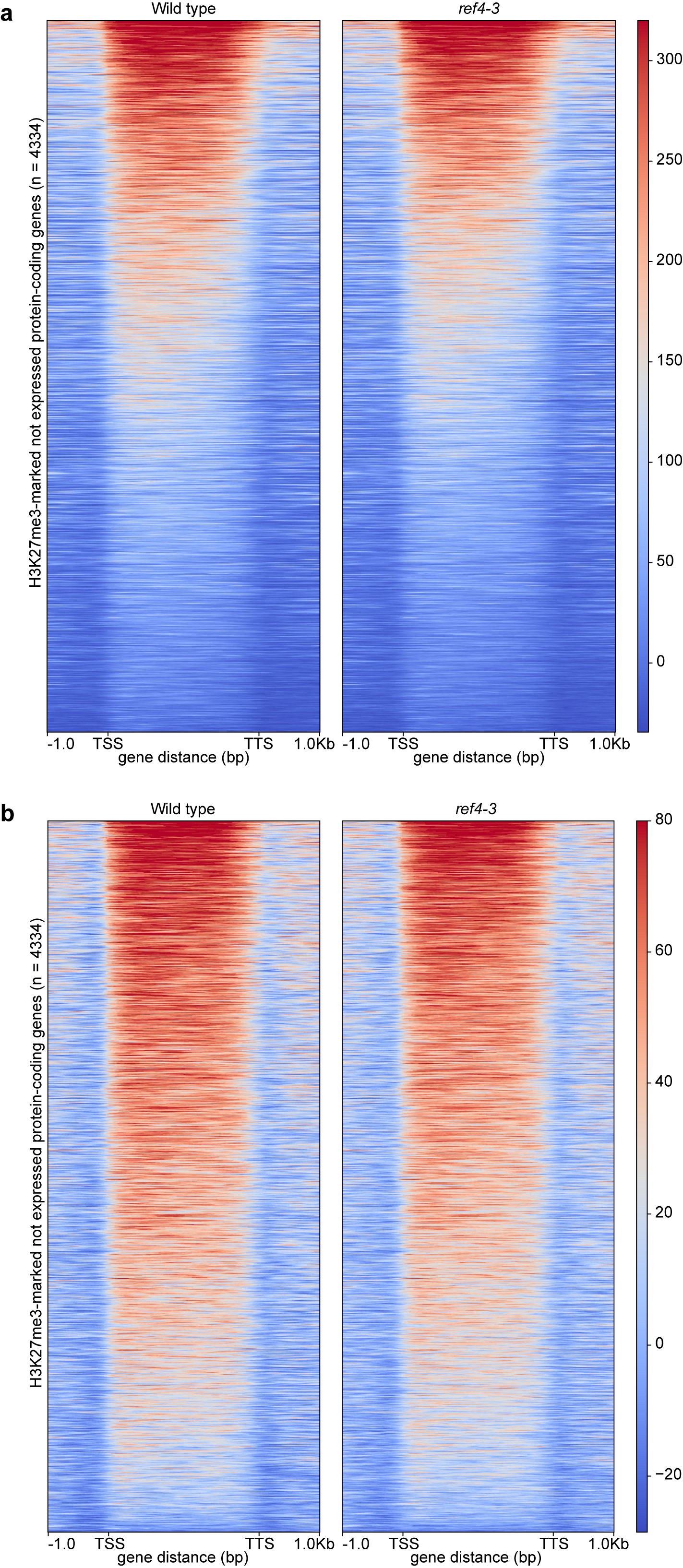

Supplement: jkae301_Supplementary_Data [file jkae301_supplementary_data.zip › Figure_S4_G3-2024-405531.tif]

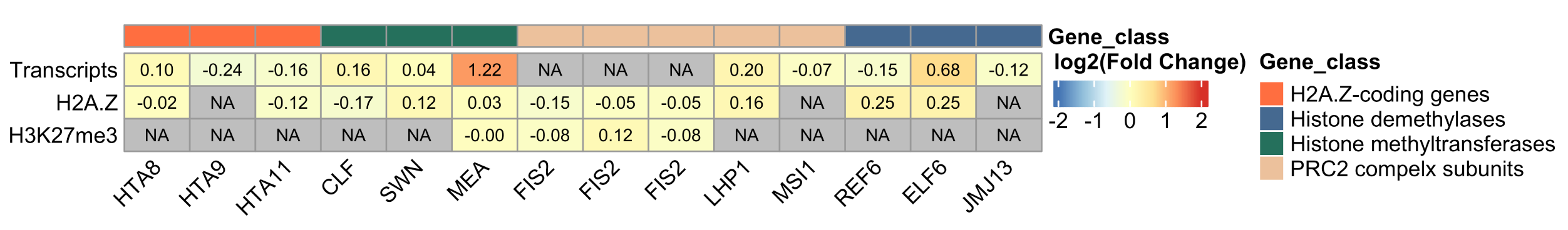

Supplement: jkae301_Supplementary_Data [file jkae301_supplementary_data.zip › Figure_S5_G3-2024-405531.tif]

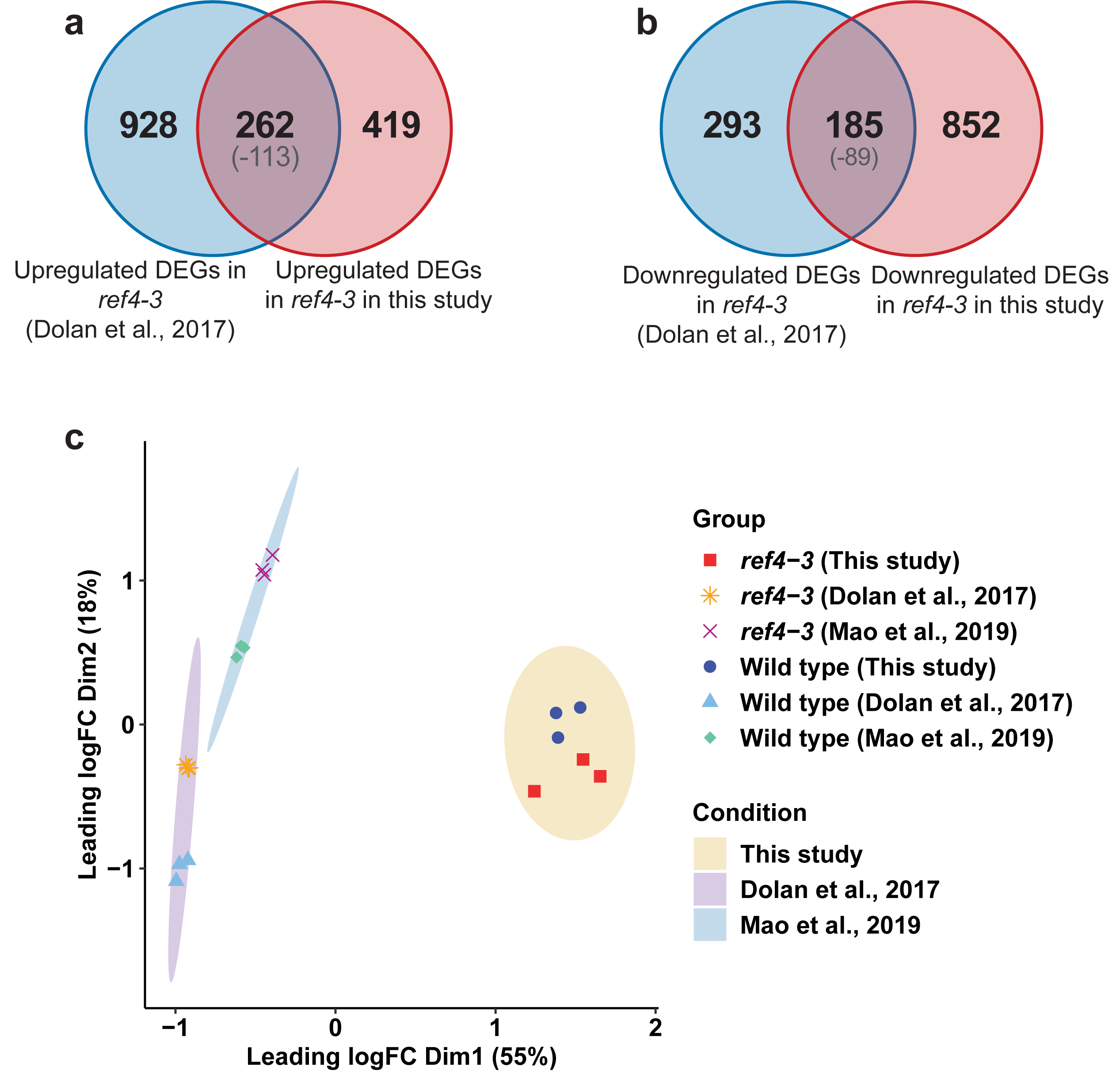

Supplement: jkae301_Supplementary_Data [file jkae301_supplementary_data.zip › Figure_S6_G3-2024-405531.tif]
